# Supplementary figures and images for: The dual action of human antibodies specific to Plasmodium falciparum PfRH5 and PfCyRPA: Blocking invasion and inactivating extracellular merozoites
Source: PLoS Pathog. 2023 Sep 15;19(9):e1011182. doi: 10.1371/journal.ppat.1011182 (PMC10529537; doi:10.1371/journal.ppat.1011182)

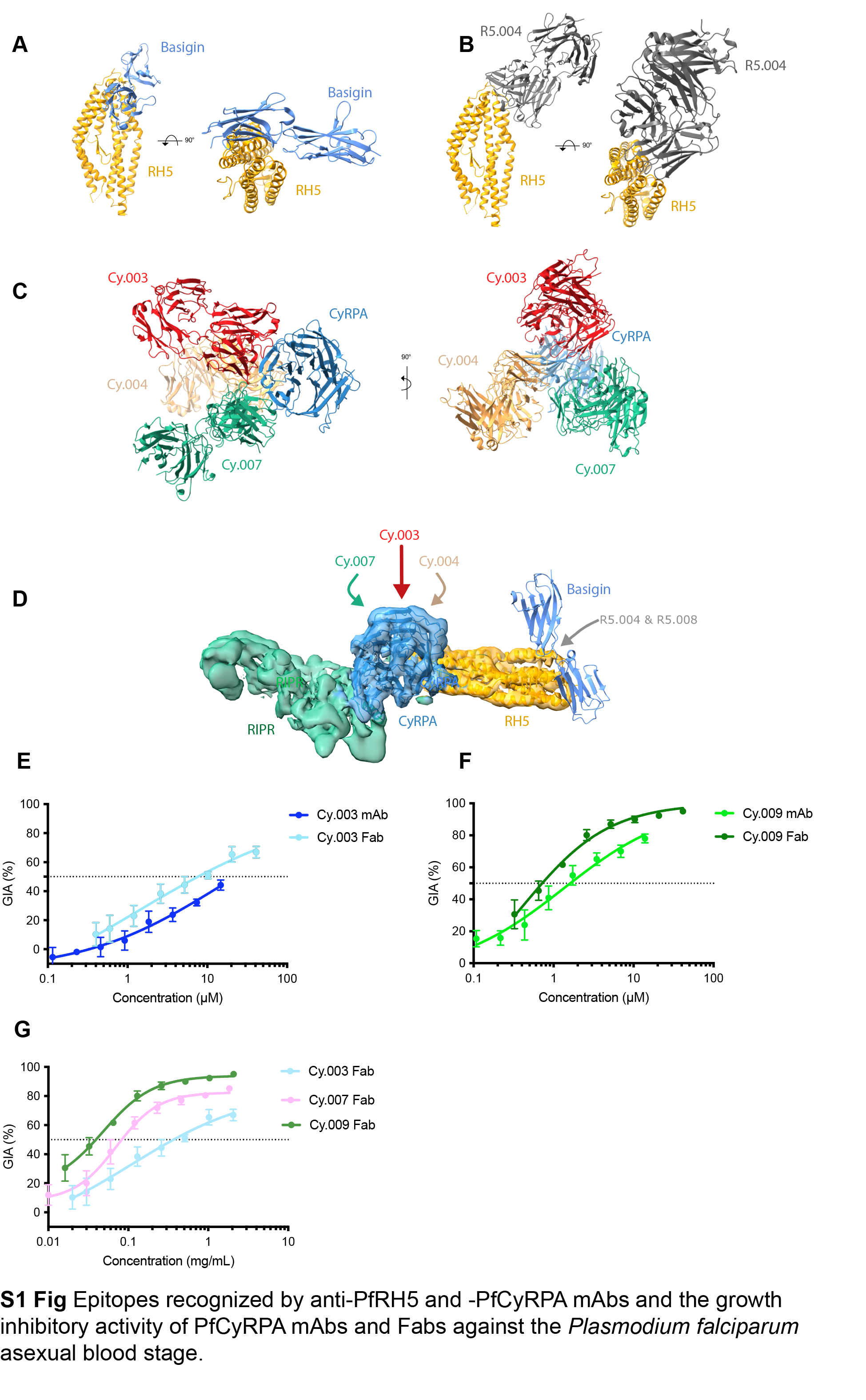

Supplement: S1 Fig — (A) Crystal structure of PfRH5 bound to basigin (protein data bank (PDB) ID: 4U0Q [45]). (B) Crystal structure of PfRH5 bound to R5.004 (PDB ID: 6RCU [4]). No structure of R5.008 is available, however both R5.004 and R5.008 compete with basigin for binding but do not compete with each other [4]. (C) Crystal structure of PfCyRPA bound to Cy.003, Cy.004, and Cy.007 (PDB ID: 7PI3 [19]). No structure is available for Cy.009 however Cy.004 and Cy.009 bind overlapping epitopes [19]. (D) Location of PfRH5 and PfCyRPA mAbs used in this study in the context of the PfRH5 (yellow), PfCyRPA (blue), PfRIPR (green) complex bound to basigin (composite image using PDB ID: 6MPV [8] & 4U0Q [45]). (E and F) Schizont stage Plasmodium falciparum 3D7 parasites were incubated with a micromolar dilution series of monoclonal antibodies (mAbs) and Fab fragments of Cy.003 and Cy.009 and grown for 40 h before parasite growth inhibitory activity (GIA) was quantified by measuring lactate dehydrogenase activity. (G) Comparison of GIA activities of anti-PfCyRPA Fabs. (TIF) [file ppat.1011182.s001.tif]

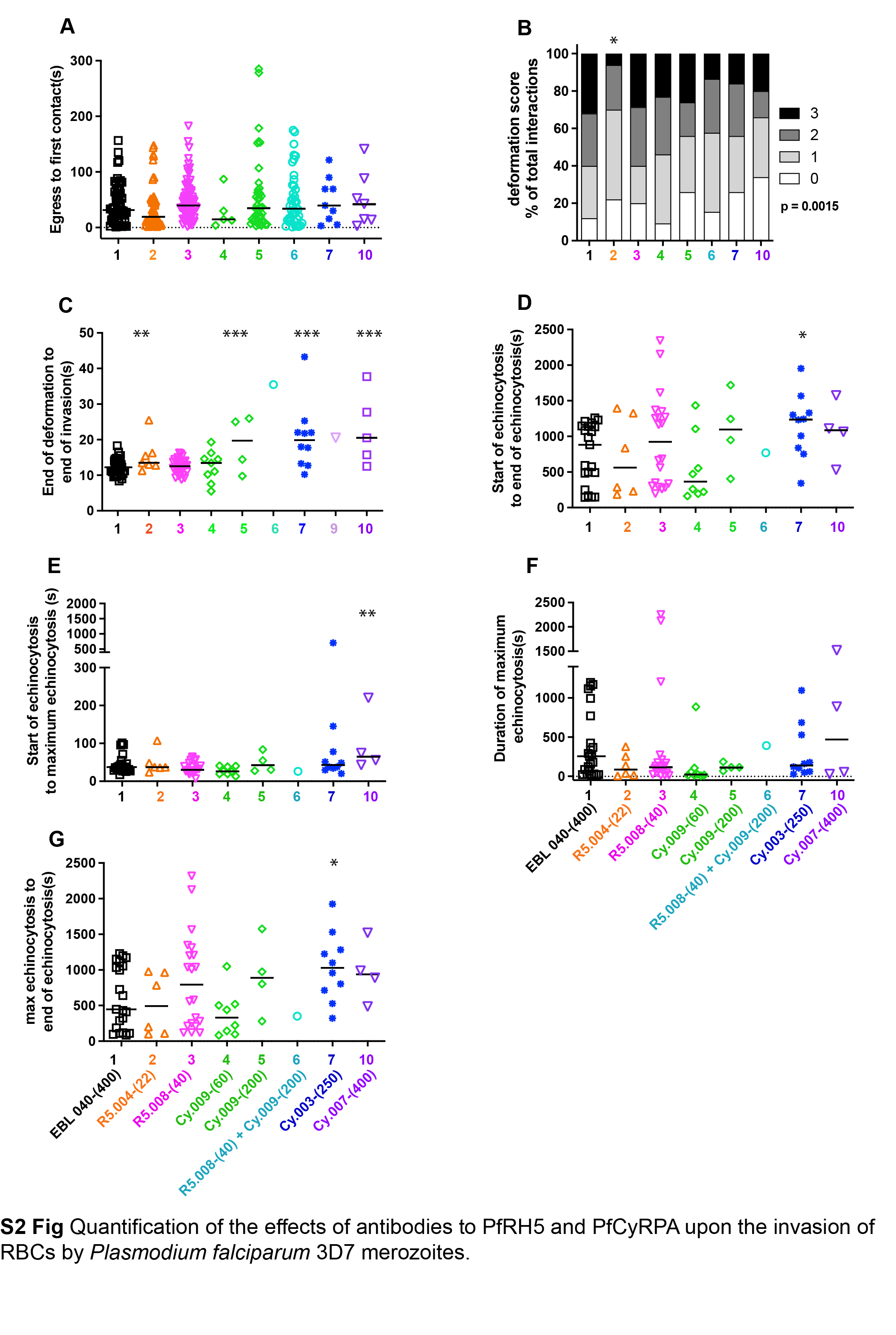

Supplement: S2 Fig — Video microscopy of several merozoite egress events was observed in the presence of antibodies with concentrations in μg/mL indicated in brackets. (A) The times from egress to first contact were not significantly different indicating the imaging conditions were consistent. (B) The degree of deformation of merozoites on erythrocyte surfaces was quantified according to [20] in the presence of antibodies. R5.004-(22) caused significantly less deformation than the control or parasite antibodies using chi-squared analysis. (C-G) The timings of other invasion stage as indicated on the y-axes were measured using the antibody combinations names and concentrations (μg/mL) indicated below the x-axes. Antibody 9 is Cy.007 Fab-(400). Each event measured is represented by a symbol with bars indicating the median. Statistical analyses were performed using unpaired t tests in GraphPad Prism V 9.0. The asterisks indicate where parasite mAbs have altered the number of events significantly from the EBL 040 control with *p<0.05, **p<0.01 and ***p<0.001. (TIF) [file ppat.1011182.s002.tif]

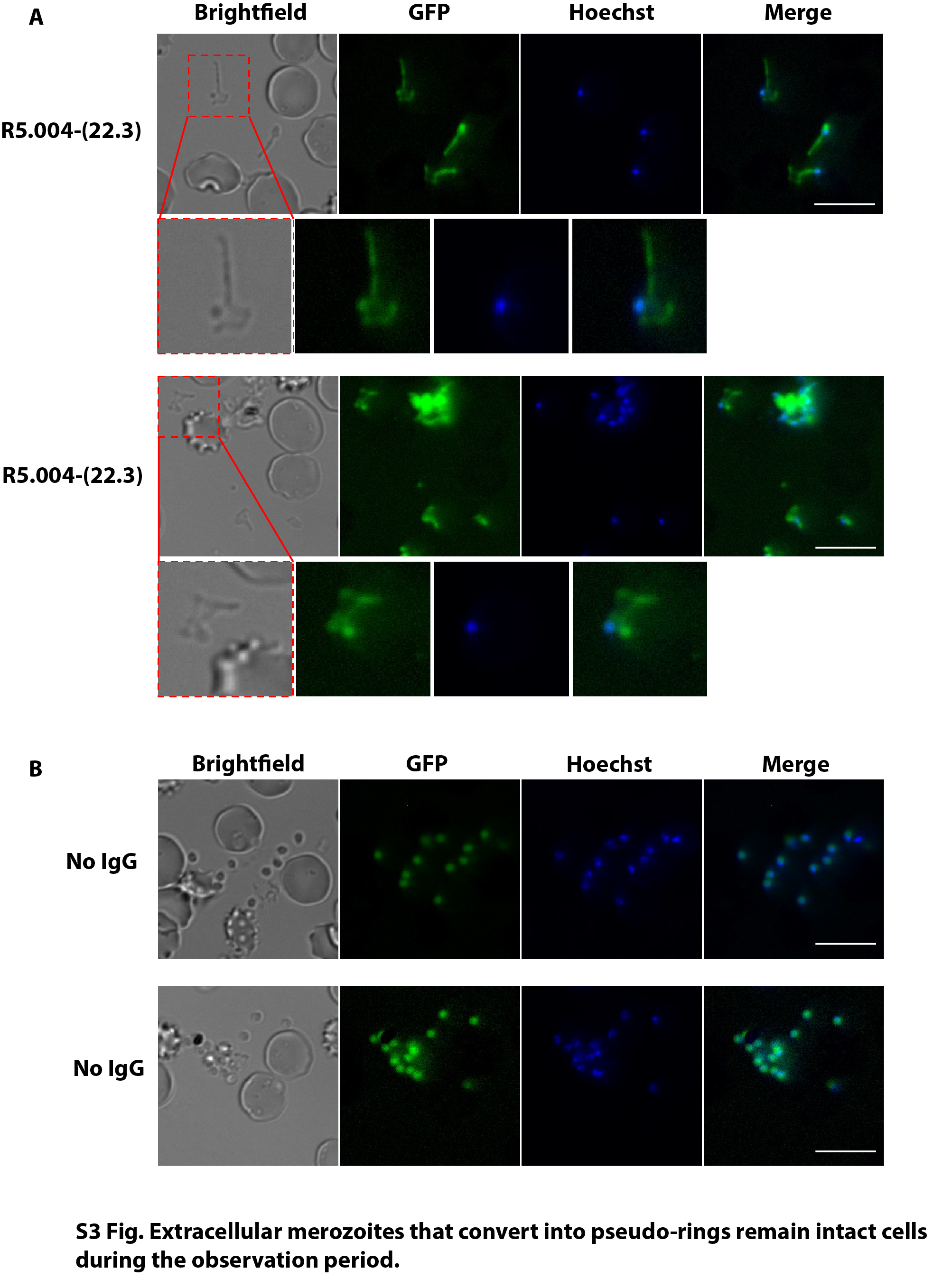

Supplement: S3 Fig — (A) Late-stage schizonts expressing cytoplasmic GFP were treated with R5.004 at 22 μg/mL. After the merozoites had egressed and begun to form pseudo-rings they were imaged and were found to have retained their GFP fluorescence indicating the cells were likely intact as loss of cytoplasmic integrity would have greatly reduced their fluorescence. Selected pseudo-rings have been enlarged as indicated with red boxes to show the pseudo-rings are intact and retain a nucleus. (B) Merozoites treated with no IgG largely retained their ovoid merozoite shapes and remained intact after egress. Parasite nuclei were stained with Hoechst and size bars are 10 μm. (TIF) [file ppat.1011182.s003.tif]

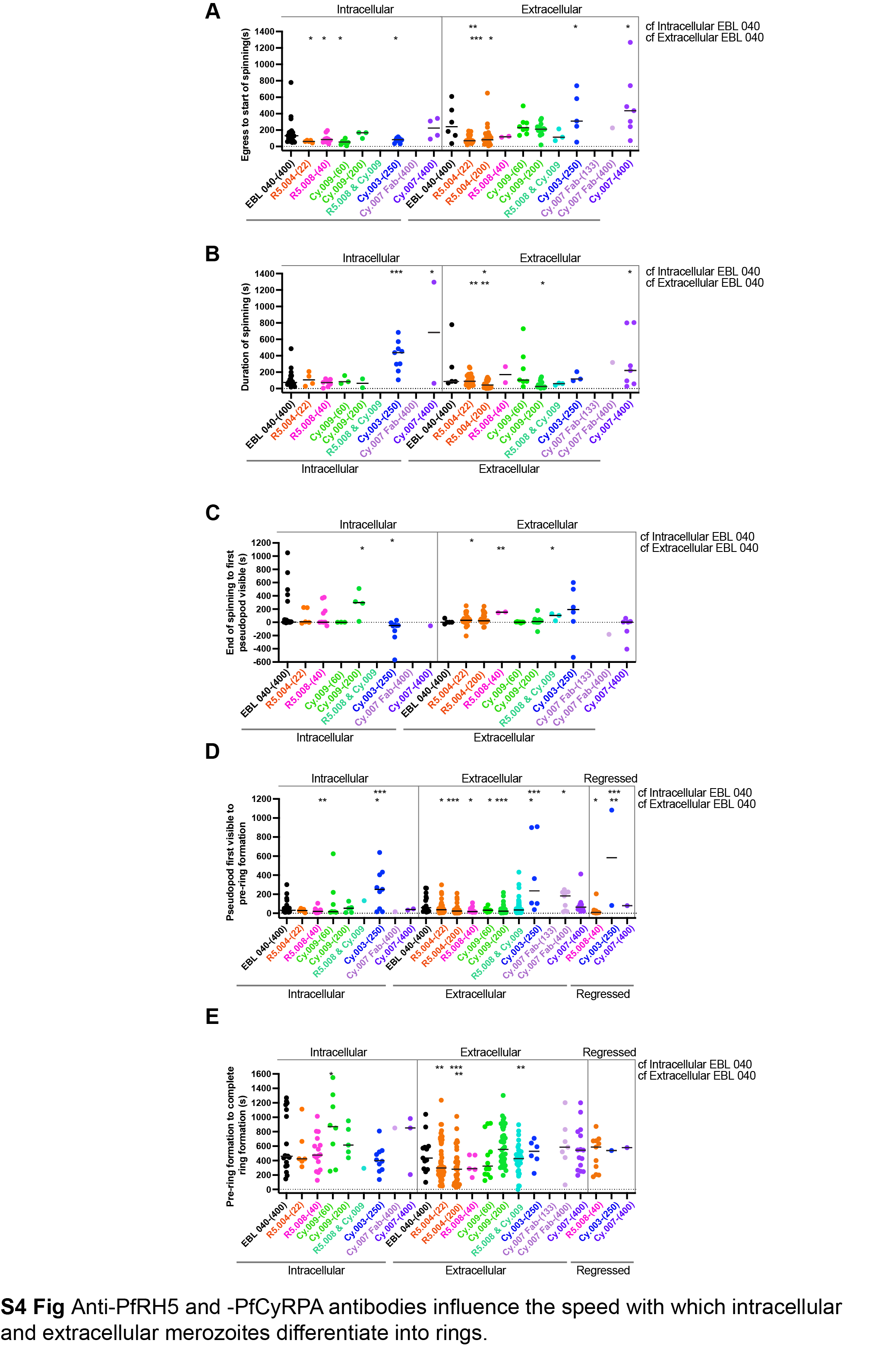

Supplement: S4 Fig — (A) The amount of time in seconds after egress before merozoites started spinning, (B) their duration of spinning, (C) time from the end of spinning to first pseudopod becoming visible, (D) time from pseudopod formation to pre-ring formation (where merozoite becomes irregularly shaped), and (E) time from pre-ring formation to complete ring formation (where parasite becomes amoeboid), were measured from live cell videos of cultured Plasmodium falciparum. Antibody names and concentrations (μg/mL) are indicated below bottom graph. Each event measured is represented by a symbol and bars indicate the medians. Statistical analyses were performed using unpaired t tests in GraphPad Prism V 9.0. The asterisks indicate where parasite mAbs have altered the number of events significantly from the intracellular and extracellular EBL 040 controls with *p<0.05, **p<0.01 and ***p<0.001. (TIF) [file ppat.1011182.s004.tif]
